# Self-Assessment of Addiction Medicine Core Competencies in Four Year Groups of Psychiatrists in Training: Efficacy of the Addiction Medicine Training Needs Assessment Scale in a Local Training Context

**DOI:** 10.1159/000528409

**Published:** 2023-01-17

**Authors:** W.J. Lucas Pinxten, Darius Jokūbonis, Virginija Adomaitiene, Darius Leskauskas, Giel J.M. Hutschemaekers, Cornelis A.J. De Jong

**Affiliations:** ^a^Behavioral Science Institute, Radboud University, Nijmegen, The Netherlands; ^b^Faculty of Psychology and Neuroscience, Department of Work and Social Psychology, Maastricht University, Maastricht, The Netherlands; ^c^Department of Psychiatry of the Lithuanian University of Health Sciences, Kaunas, Lithuania

**Keywords:** Self-assessment, Addiction medicine, Core competencies, Self-regulated learning and curriculum development

## Abstract

**Background:**

In addiction medicine training, self-assessment is increasingly used to support self-regulation learning by identifying standards of excellence, competence gaps, and training needs. To ensure psychiatrists in Lithuania also develop specific addiction competencies, the Lithuanian Health Sciences University faculty in Kaunas developed an addiction psychiatry curriculum.

**Objectives:**

The aim of this research is to explore the efficacy of the AM-TNA scale to measure individual and group differences in proficiency in the core competencies of addiction medicine. A cross-sectional study and a convenience sample were used.

**Method:**

We studied the differences in performance in addiction medicine competencies between 4 successive year groups and analysed the variance to determine the statistical differences between the means of 4 year groups with biases, resulting from repeated measurement statistically corrected-for.

**Results:**

Of the psychiatrists in training, 41% or 59% completed the scale. The assessment of competencies suggested that all but 2 competencies differ significantly (*p* < 0.05) between the 4 groups. The post hoc analyses indicated that mean scores for 24 of the 30 core competencies differed significantly between the year groups (*p* < 0.05) and showed a gradual increase in scores of self-assessed competencies over the 4 year groups. We found adequate scale variance and a gradual increase in self-assessed competencies between the 4 year groups, suggesting a positive association between the results of incremental professional training and improved self-assessed substance use disorders (SUD) competency scores.

**Conclusions:**

This study illustrates the efficacy of the AM-TNA scale as an assessment instrument in a local training context. Future research should aim to have larger sample sizes, be longitudinal in design, assess individual progress, and focus on comparing and combining self-reported competencies with validated objective external assessment and feedback.

## Introduction

### Lifelong Self-Regulated Learning and Self-Assessment

Substance use disorders (SUD) are a major contributor to the global burden of disease, ranking fifth in the top-10 causes of disability and death and a major cause of health care expenditure [1, 2], requiring not only improvement of effectiveness and organization of addiction medicine training across the globe [3], but also adequate postgraduate and continuous medical education for addiction medicine specialists.

In order to stay abreast of the latest developments and to strengthen professional expertise and competence, addiction specialists are required to be successful lifelong self-regulated learners: being able to identify their own strengths and weaknesses and having effective self-assessment and improvement strategies and tools [4]. Self-assessment can be used as an explicit way to strengthen formative assessment and is, in established addiction medicine curricula, increasingly used to support self-regulation learning [5]. However, stand-alone self-assessment has, especially when used as summative evaluation for medical students, accuracy limitations [6]. In order to encourage the use of valid self-assessment tools by addiction professionals, we chose to study self-assessment from the perspective of learning experience and as a formative assessment tool. We also stress the importance of combining self-assessment with other forms of assessment, such as feedback by peers, senior colleagues, preferably using multiple assessment tools [7, 8, 9].

### Addiction, Training, and Assessment in Lithuania

In Lithuania, alcohol use contributes to 25% and smoking to 14% of total deaths [10]. The Lithuanian SUD-services are the responsibility of psychiatrists, who study SUD as a common comorbidity of other psychiatric conditions during their 4-year general psychiatry residential training. In order to ensure these trainees also develop specific SUD competencies, the Kaunas Lithuanian University of Health Sciences (LUHS) developed a specific addiction psychiatry curriculum, including a 3-month supervised addiction medicine placement in the final 2 years. Progress on SUD competencies is assessed using the Lithuanian version of the Addiction Medicine Training Needs Assessment scale (AM-TNA) [11]. This self-assessment instrument is useful for connecting addiction medicine training needs to the content of the training courses, through supporting self-regulated learning by both generalists and specialists. As a result, the aim of this study is to explore the AM-TNA's self-assessment efficacy to identify both individual and group differences in strengths and weaknesses in core competencies among 4 successive year groups (YG) of psychiatrists in training enrolled in the aforementioned addiction psychiatry course.

### Objectives

- Exploring the efficacy of the AM-TNA scale in assessing individual differences in proficiency in addiction competencies.
- Exploring the association (if any) between the AM-TNA self-assessment scores and the level of postgraduate training groups (years 1–4) of psychiatrists in training.

## Materials and Methods

### Design

In this preliminary evaluation of self-assessed addiction medicine core competencies and training, a cross-sectional comparison was chosen to measure differences in perceived proficiency of 30 core addiction competencies, in 4 successive YG of psychiatrists in training at Kaunas LUHS.

### Sample and Participants

To measures differences in training outcomes, we used a convenience sample of all psychiatrists in training at the Kaunas LUHS psychiatry clinic in May 2019. The Lithuanian training capacity for psychiatrists is around 100: being the responsibility of the medical schools of the Universities of Vilnius and Kaunas.

### Ethical Considerations

The study complies with the Declaration of Helsinki: written informed consent from the participating psychiatrists in training was established before the completion of the anonymous questionnaire.

### The Instrument

The AM-TNA was developed to measure progress and gaps on addiction medicine competencies in Indonesia. The instrument consists of a 30-item questionnaire containing self-reported proficiency on internationally recognized addiction medicine specific core competencies, using a 5-point Likert-scale ranging from not-at-all proficient-fully proficient. The instrument assesses 21 clinical and 9 non-clinical competencies in 3 professional domains: assessment and diagnosis, starting treatment, and maintaining treatment [12]. The Indonesian AM-TNA scale was translated into Dutch, Lithuanian, and English. The scale has besides a proven reliability and internal validity a clear-cut 2-dimensional factor structure [11].

### Analysis

To demonstrate psychiatrists in training can self-assess their proficiency in addiction competencies using the AM-TNA scale we expect, besides a high response rate, very few missing values, an adequate variance in results, and a significant difference in means between the 4 YG. To assess the relationship between these groups and self-assessed competencies, an a priori analysis of variance (ANOVA) is planned. To test the mean differences of 30 competencies between the 4 YG, a between-subject test will be used.

To explore the association between the AM-TNA self-assessment scores and the level of postgraduate training (YG 1–4), we expect that mean scores will gradually increase over the years. In order to correct for the risk of false positives resulting from repeated measurement and to limit the instance of false positive results (Type-I errors), we will execute a Bonferroni-adjusted post hoc analysis: a series of *t* tests on each pair of classes using a corrected *p* value.

## Results

All 52 participants received the AM-TNA scale, of which 41 questionnaires (79%) were completed by 4 YG: YG 1:14, YG 2:8, YG 3:10, and YG 4:9. Mean age, respectively: 26, 26, 32, and 29, *M* = 28.29 (*SD*: 4.627). Gender: 29 (70.7%) female and 12 (29.3%) male. There were no missing values. The sum scores of 28 competencies differed significantly between the 4 YG (*p* < 0.05) with the exception of the competencies 6 and 10 (Table 1, Column 8).

**Fig. 1:**
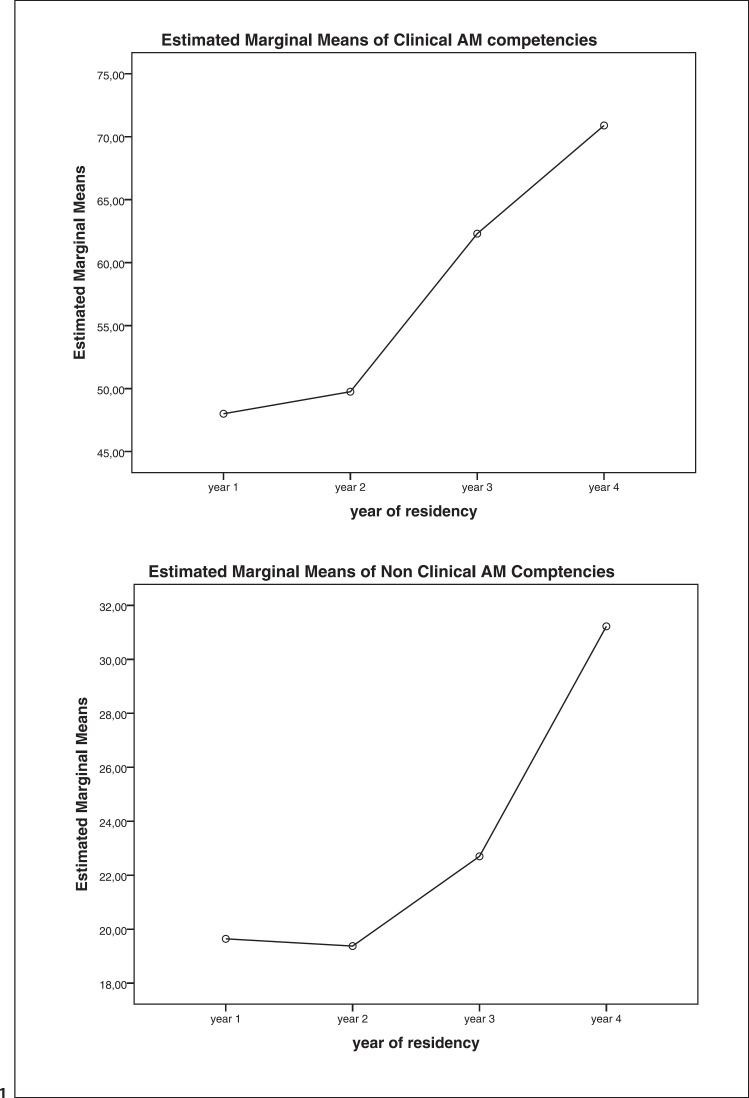
Bonferroni plots: estimated marginal means (*y*-axis) of combined clinical addiction medicine competencies (top) and combined non-clinical Competences (bottom) against 4 year groups of residential psychiatry specialists in training (*x*-axis).

All competencies except # 6 had *F* values well above 1, indicating that in-between group variance was much more important than with-in group variance. The Bonferroni-adjusted post hoc analysis of 6 pairs and 4 YG is presented in Table 1. Bonferroni correction was α/*n* = 0.05/6 = *p*< 0.008 indicates that the mean scores of 19 of the 30 competencies differ significantly between one or more training years (Table 1, column 2). 11 clinical competencies (1, 2, 3, 6, 7, 10, 12, 15, 16, 18, 20) do not show significant statistical differences between the training groups, whilst all competencies but 6 and 10 showed a tendency in the predicted direction. Comparing the total of the clinical and non-clinical competencies resulted in significant differences (*p* < 0.001) between the 4 YG (resp. 1 < 4, 2 < 4 and 1 < 4, 2 < 4, 3 < 4). The gradual increase in marginal means of the combined clinical and non-clinical competencies over the 4 classes in training is visualized in Figure 1.

## Conclusion

The cross-sectional study design fitted the preliminary evaluation need and the sample represented around 50% the psychiatrists in training in Lithuania. We found a significant difference in mean scores between the 4 YG of psychiatrists in training. This indicates that the AM-TNA scale can measure systematic group differences in perceived proficiency in the SUD core competencies.

Furthermore, an excellent response rate (79%) and no missing values supported the efficacy of the AM-TNA scale (Objective 1). The Bonferroni plots illustrated a gradual increase in marginal means of the combined clinical and non-clinical competencies in the 4 training groups. This suggests an association between the AM-TNA scale self-assessed learning (vertical axis) and cumulative training years (horizontal axis) (Objective 2).

This explorative, cross-sectional study has limitations. The results are based on a small convenience sample, and recruiting respondents from a single training location (LUHS) may have introduced some selection bias into our study. The study design choice also means we cannot determine causality.

This study clearly confirms the relationship, between AM-TNA scale self-assessment scores and the number of years spent in psychiatry training. We may have missed some individual variability and some individual self-assessment of proficiency in SUD competencies may have plateaued or even declined. Finally, the AM-TNA is a self-reported scale, reflecting the respondents' perceived competency and not their objectively measured skills. This limitation would be concerning if self-ratings in the YG 1 were unusually high as it may indicate socially desirable responses. However, this was not detected in our study.

Competency-based assessment is gold standard in medical training. It is also worthwhile to note that self-assessment of medical competencies, despite its accuracy limitations, is an important component of lifelong professional learning, like continuous medical education and an established tool to promote both academic achievement and self-regulated medical learning [13].

Validated self-assessment tools are particularly useful in encouraging self-assessment of addiction caregivers' perceived SUD competencies, and as such stimulate the acquisition of addiction skills by specialized and non-specialized professionals. Despite the study's limitations, the AM-TNA self-assessment scale measured individual proficiency and produced significant group differences in perceived SUD competencies in a small local training context.

Accuracy and external validity issues of self-assessment scales like the AM-TNA can be overcome when used as formative evaluation tools in combination and in support of external and objective evaluations and observations, critical appraisal, reflection reports. This study illustrates that the self-assessment AM-TNA scale can measure individual proficiency and significant incremental differences between mean scores of perceived proficiencies in addiction medicine core competencies in a 4 YG psychiatry training setting. We suggest it could also function well in larger-scale training settings. The AM-TNA scale might also support individual self-regulated learning and could be helpful to improve individual competencies. Future research should aim to investigate differences in larger sample sizes over time, assess individual progress, and focus on comparing and combining self-reported competencies with validated objective external assessments of learning, attitudes, and clinical practice.

## Statement of Ethics

The research was conducted ethically in accordance with the World Medical Association Declaration of Helsinki. Study approval: the study protocol was reviewed and approved by the Kaunas Lithuanian University of Health Sciences Ethics Committee: approval number Nr-BC-LSMU-121. Participation consent: written consent from participating psychiatry specialists in training to participate in the study was obtained before completion of the anonymous questionnaire.

## Conflict of Interest Statement

The authors report no conflicts of interest. The authors alone are responsible for the content and writing of the publication.

## Funding Sources

This research received no specific grant from any funding agency in the public, commercial, or not-for-profit sectors.

## Author Contributions

Lucas Pinxten: corresponding author, research conception and design, data collection, contributed to data analysis and interpretation of results, writing, revising, and finalizing the manuscript. Darius Jokūbonis: contributed to data collection, interpretation of results, revising, and approving the manuscript. Virginija Adomaitiene and Darius Leskauskas: contributed to the interpretation of the results, revising, and approving the manuscript. Giel Hutschemaekers: contributed to the research design, the interpretation of the results, writing, revising, and approving the manuscript. Cornelis de Jong: contributed to research conception and design, data collection, analysis, and interpretation, writing, revising, and approving the manuscript.

## Data Availability Statement

After publication of this article, all participating psychiatrist in training will receive a copy of this article and the university research protocol/ethics clearance (Nr-BC-LSMU-121) from the authors related to the Kaunas Lithuanian University of Health Sciences. On request, the corresponding author will share the anonymized raw data set and the SPSS syntax and output after approval by the Kaunas Lithuanian University of Health Sciences.

## Figures and Tables

**Table 1:** Mean addiction medicine competencies of 4 YG of psychiatrists in training

Dependent variable: core AM competences over three domains	Sign	Year 1, mean (SD)	Year 2, mean (SD)	Year 3, mean (SD)	Year 4, mean (SD)	*F*	*p* value, 0.008	Comp. 1–2, 3–4
*Assessment and diagnosis*								
01. Selecting appropriate screening or assessment tools for substance use	NS	1.93 (0.48)	2.13(0.99)	2.70 (0.68)	3.00(1.12)	4.052	0.014	1 < 4
02. Screening risk of substance use problems	NS	2.14(0.14)	2.38 (0.92)	3.00 (0.82)	2.89 (93)	3.384	0.028	
03. Assessing substance use problems by taking a patients history	NS	2.57 (0.65)	3.38(1.06)	3.46 (0.70)	3.33(71)	3.356	0.029	1 < 4
04. Assessing substance use problems by a physical examination	X	2.14(0.53)	2.50 (0.93)	3.00 (0.94)	3.44(1.01)	5.017	0.005	
05. Selecting appropriate diagnostic laboratory tests	X	2.57 (0.65)	2.75 (0.71)	2.50 (0.53)	3.67 (0.87)	5.927	0.002	1 < 3, 1 < 4
06. Interpreting substance abuse using screening, assessment, laboratory results	NS	2.71 (0.61)	2.75 (0.71)	2.90 (0.57)	3.22(1.09)	0.935	0.434	
07. Using evidence-based approach in assessment	NS	1.86 (0.53)	1.75 (0.46)	2.40 (0.84)	2.56 (0.88)	3.149	0.036	

*Starting treatment*								
08. Formulating a diagnose using standard diagnostic criteria for addiction disorders (DSM or ICD)	X	2.64 (0.84)	3.25(0.71)	3.60 (0.52)	3.67 (0.87)	4.620	0.008	1 < 3, 1 < 4
09. Explaining diagnose and prevention and treatment plan to the patient	X	2.43 (0.65)	2.38 (0.74)	3.30 (0.48)	3.56 (0.88)	7.532	0.000	1 < 3, 1 < 4
10. Developing a written treatment plan	NS	2.36(1.01)	2.38 (0.74)	2.50 (0.85)	3.22 (1.20)	1.687	0.187	
11. Selecting indicated initial treatment medications	X	2.21 (0.58)	2.13(0.84)	3.00 (0.67)	3.56 (0.88)	8.510	<0.001	1 < 4, 2 < 4
12. Starting maintenance and substitution treatment	NS	1.93 (0.73)	2.09 (0.93)	2.80 (0.79)	2.78(1.20)	2.925	0.046	
13. Providing general medical and social care to addiction patient	X	2.21 (0.70)	1.75 (0.71)	2.40 (0.84)	3.33(1.00)	5.946	0.002	1 < 4, 2 < 4
14. Using evidence-based and up-to-date approach in treatment	X	2.00 (0.88)	1.63 (0.52)	2.40 (0.84)	3.11 (0.93)	5.335	0.004	1 < 4, 2 < 4
15. Using motivational techniques to support adherence to treatment	NS	2.07(1.90)	1.63 (0.52)	2.30(1.06)	2.89 (0.78)	2.994	0.043	2 < 4
16. Using basic psychosocial strategies to support recovery	NS	1.86 (0.95)	1.63 (0.52)	2.30 (0.95)	3.00 (0.87)	4.506	0.009	1 < 4, 2 < 4
17. Consulting other medical professionals	X	2.57(0.51)	2.63 (0.92)	3.10(0.88)	3.89(1.05)	5.361	0.004	1 < 4, 2 < 4
18. Consulting non-medical professionals	NS	2.71 (0.91)	2.25(1.17)	2.40 (0.97)	2.50 (0.85)	3.625	0.022	2 < 4

*Managing treatment*								
19. Selecting indicated maintenance and treatment medications	X	2.00 (0.68)	2.50(1.20)	2.50 (0.85)	3.56 (0.88)	9.606	<0.001	1 < 4, 2 < 4, 3 < 4
20. Managing intoxication	NS	2.36 (0.75)	2.50(1.20)	3.00 (0.67)	3.56 (0.88)	4.047	0.014	1 < 4
21. Managing withdrawal	X	2.50 (0.94)	2.88(1.13)	3.60 (0.97)	3.44 (0.73)	3.328	0.030	1 < 3
22. Managing craving	X	1.86 (0.95)	1.38 (0.52)	2.70 (0.82)	2.67(0.71)	5.952	0.002	2 < 3, 2 < 4
23. Managing overdoses	X	1.43 (0.51)	1.88 (0.99)	2.30 (0.68)	2.89(1.05)	6.677	0.001	1 < 4
24. Managing medical emergencies	NS	1.93 (0.48)	2.50(1.20)	3.00 (0.94)	3.00 (0.71)	4.585	0.008	1 < 3.1 <4
25. Monitoring substance use patients for relapse throughout treatment	X	1.57(0.51)	2.00(1.07)	2.80 (0.92)	3.33(1.00)	9.213	0.000	1 < 3, 1 < 4
26. Using groups interventions effectively	X	1.43 (0.51)	1.38 (0.52)	1.80 (0.92)	1.78 (0.92)	7.222	0.001	1 < 4, 2 < 4
27 Collaborating with other medical professionals	X	2.36 (0.84)	2.38 (0.92)	2.60(1.08)	3.89 (0.60)	6.599	0.001	1 < 4, 2 < 4, 3 < 4
28. Collaborating with non-medical professionals	X	2.36 (0.93)	2.25(1.17)	2.10(1.20)	3.78 (0.67)	5.568	0.003	1 < 4, 2 < 4, 3 < 4
29. Distinguishing substance abuse problems from psychiatric comorbidity	X	2.14(0.36)	2.25(1.17)	3.00 (0.67)	3.56 (0.53)	9.508	<0.001	1 < 4, 2 < 4
30. Addressing additional psychological and psychiatric problems	X	2.21 (0.43)	2.63(1.06)	3.00 (0.82)	3.89 (0.60)	10.32	<0.001	1 < 4, 2 < 4
Clinical addiction medicine competencies	X	48.00 (8.36)	49.75(12.9)	62.30(10.8)	70.89(14.3)	9.21	<0.001	1 < 4, 2 < 4
Non-clinical addiction medicine competencies	X	19.64(3.67)	19.38(5.24)	22.70 (6.33)	31.22(5.19)	11.40	<0.001	1 < 4, 2 < 4, 3 < 4
